# The Book World of Medicine and Science

**Published:** 1907-06-08

**Authors:** 


### The Book World of Medicine and Science.

Medical Philosophy. (?) By W. Russell. Published by Henry Kimpton, London, and Alexander Stenhouse, Glasgow. 1907. Pp. xvi.+504. Price 21s. net.

The author believes that two propositions do not now receive proper respect from the medical profession. They are, that the origin of all disease whatsoever is a humour caused by indigestion, and that indigestion is always curable by purgation. If these premisses are absolutely infallible, as he is convinced, his conclusion is logical enough that every illness, local or general, demands purgatives, and purgatives only. In support of this theme he presents a mass of quotations from the works of medical and other authors. These constitute about 95 per cent. of the text, and of them a similar proportion date back half a century or more. They are culled from a field wide enough to include Kingsley and the *Family Herald*, Goldsmith (misquoted by the by) and Rider Haggard, Hippocrates and the *Family Doctor*, the Bible and *Lloyd's*! About half the chapters begin with the same short summary of the argument; but, lest this should pall with repetition, the punctuation is ingeniously varied so that no two versions are actually the same. We cannot refrain from quoting part of the last sentence: "The other so-called remedies of man . . . diuretics . . . massage . . . local applications, etc., are unworthy of mention; . . . in showing that others have condemned them I should only be wasting your time, which might be more profitably employed in studying the other parts of this work."

